# Medical Student Perspectives on the Future of Psychiatry: The View from Turkey

**DOI:** 10.1192/j.eurpsy.2022.191

**Published:** 2022-09-01

**Authors:** U. Kahraman, O. Kilic

**Affiliations:** 1 Bezmialem Vakif University, Faculty Of Medicine, Istanbul, Turkey; 2 Bezmialem Vakif University Faculty of Medicine, Department Of Psychiatry, Istanbul, Turkey

**Keywords:** future of psychiatry, perspective, medical student

## Abstract

Psychiatry is still considerably ‘young’ compared to other positive sciences. Thus, it holds a huge potential for improvement of the current diagnostic and classification systems and modes of treatment particularly. For instance, the Research Domain Criteria Project will certainly generate novel research questions that will shed a light upon mechanisms, and processes for the expression of psychiatric phenomenology and develop psychiatric treatments. The new era of Digital Psychiatry/Telepsychiatry and real-time mobile monitoring are other approaches that have a lot to offer to advance the field of psychiatry. Despite these developments, the stigma around psychiatry is still a big obstacle to tackle with. Addressing and reducing stigma during medical education should benefit from training and experience co-facilitated by people living with mental illness ideally starting from the early years in medical school. Besides clinic rotations, student clubs, student scientific congresses, and clinical research that facilitate contact with the patients may be potential platforms to attract medical students’ attention to the work of psychiatry. The speaker will touch upon some examples and implementations from the medical schools in Turkey.

**Disclosure:**

No significant relationships.

